# Acridine as an Anti-Tumour Agent: A Critical Review

**DOI:** 10.3390/molecules28010193

**Published:** 2022-12-26

**Authors:** Potlapati Varakumar, Kalirajan Rajagopal, Baliwada Aparna, Kannan Raman, Gowramma Byran, Clara Mariana Gonçalves Lima, Salma Rashid, Mohammed H. Nafady, Talha Bin Emran, Sławomir Wybraniec

**Affiliations:** 1 Department of Pharmaceutical Chemistry, JSS College of Pharmacy (JSS Academy of Higher Education & Research), Ooty 643001, India; 2 Department of Food Science, Federal University of Lavras, Lavras 37200-900, Brazil; 3 Department of Pharmacy, Faculty of Allied Health Sciences, Daffodil International University, Dhaka 1207, Bangladesh; 4 Faculty of Applied Health Science Technology, Misr University for Science and Technology, Giza 12568, Egypt; 5 Department of Pharmacy, BGC Trust University Bangladesh, Chittagong 4381, Bangladesh; 6 Department of Chemical Technology and Environmental Analysis, Faculty of Chemical Engineering and Technology, Cracow University of Technology, Warszawska 24, 31-155 Krakow, Poland

**Keywords:** acridine, anti-cancer, cancer cell lines, DNA-intercalation, topoisomerase, in vitro assay

## Abstract

This review summarized the current breakthroughs in the chemistry of acridines as anti-cancer agents, including new structural and biologically active acridine attributes. Acridine derivatives are a class of compounds that are being extensively researched as potential anti-cancer drugs. Acridines are well-known for their high cytotoxic activity; however, their clinical application is restricted or even excluded as a result of side effects. The photocytotoxicity of propyl acridine acts against leukaemia cell lines, with C1748 being a promising anti-tumour drug against UDP-UGT’s. CK0403 is reported in breast cancer treatment and is more potent than CK0402 against estrogen receptor-negative HER2. Acridine platinum (Pt) complexes have shown specificity on the evaluated DNA sequences; 9-anilinoacridine core, which intercalates DNA, and a methyl triazene DNA-methylating moiety were also studied. Acridine thiourea gold and acridinone derivatives act against cell lines such as MDA-MB-231, SK-BR-3, and MCF-7. Benzimidazole acridine compounds demonstrated cytotoxic activity against Dual Topo and PARP-1. Quinacrine, thiazacridine, and azacridine are reported as anti-cancer agents, which have been reported in the previous decade and were addressed in this review article.

## 1. Introduction

Malignant tumours are commonly referred to as cancer. Malignant tumours are the world’s most serious threat to human health. Cancer is derived from the Latin word for crab. Although this is a common term, the scientific term for cancer is neoplasia, which means “new formation” in Greek. The term “malignant neoplasm” refers to a new growth that contains pathogenic or harmful features that can be seen in the body. It can destroy vital organs, as well as, in some cases, create life-threatening disruptions in the functioning of the body.

In 1870, Graebe and Caro discovered a solid, crystalline, and base compound as a type of raw, impure anthracene extracted from coal tar. The chemical was given the name acridine because of its irritating impact on the skin. Acridine is also known by the names dibenzo[b,e]pyridine,benzo[b]quinolone, 2,3,5,6-dibenzopyridine, and 10-azaanthracene. Acridine analogues have been investigated as potential cancer therapy agents, with a focus on DNA and DNA-related enzymes such as topoisomerases, telomerase, and others. Amsacrine, the most well-known anti-cancer acridine medication, applied against acute leukaemia and its derivatives can affect topoisomerase 2 and other intracellular targets in a variety of ways. One of the milestones in the history of the acridines was the development of the antitumour agents Ledakrin and Acriflavine in 1912. Quinacrine, used for apoptotic and anti-apoptotic genes, was evaluated. Several 9-aminoacridine derivatives cleave the acridine ring in the presence of primary aliphatic amines, especially in the course of solid-phase synthesis; investigations have shown that the α-amino group in peptides is a weak acridine acceptor, whereas the ε-amino group of lysine is a moderate acridine acceptor. The biological activity of acridines is mostly due to their aromatic structure planarity, which allows them to interact with the DNA structure via intercalation.

This study outlined recent breakthroughs in acridine chemical properties, with a focus on the last decade. The reactivity of the acridine ring as well as the synthesis of 9-substituted acridines as anti-cancer agents and the mechanisms of the reaction occurring at the position of 9 or 10 of 9-substituted acridines were reviewed. The potential of acridine carboxamide platinum complexes as anti-cancer agents was also addressed. As acridines constitute a class of compounds of considerable pharmaceutical interest, their biological activities were summarised.

As mentioned, this critical review discussed the current progress in the novel 9-substituted acridine heterocyclic compounds. Acridine shows various biological activities (anti-bacterial, anti-parasitic, anti-viral etc.), whereas in this review, acridine’s anti-tumour activity is particularly discussed. Additionally, to address the cytotoxicity of various acridine derivatives, IC_50_ values are given in a table.

**Figure 1:**
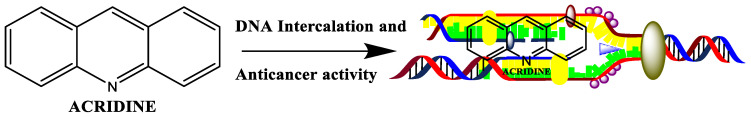
Acridine as an anti-tumour agent.

This overview is not exhaustive; instead, it highlights some recent examples of acridines, which are biologically active chemicals, and certain applications of technically or biotechnologically interesting acridines as well as various reactions on the acridine ring (Figure 1).

## 2. Acridine as an Anti-Tumour Agent

### 2.1. Acridine

**Figure 2:**
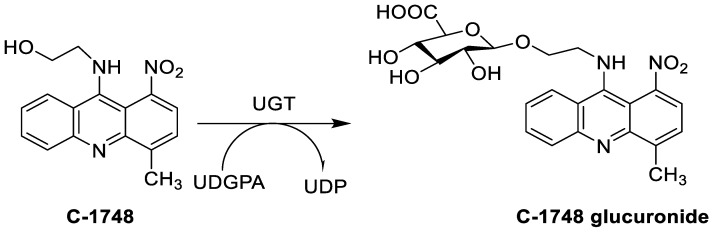
C-1748 phase I metabolic pathway.

The photocytotoxicity of propyl-AcrDTU against the murine leukaemia L1210 cell line has been reported. Previously, electron paramagnetic resonance (EPR) spectroscopy has been used to assess the formation of ROS by propyl-AcrDTU after irradiation. After confirming the production of ROS when UV-A light (>300 nm) was used to irradiate propyl-AcrDTU in the sight of molecular oxygen, to elucidate the mechanism of this chemical’s photocytotoxic effect, researchers focused on its intracellular location [1]. UV-Vis and fluorescence spectroscopy were used to synthesise and characterise two new tetrandrine-based receptors, as well as their bonding properties towards a range of nucleotides and ds-DNA, in water at pH = 7.2. In an intercalation-binding mode, two receptors had a strong affinity (K 105 M-1) and specificity of sequence for ds-DNA. This research included molecular modelling and single-crystal X-ray diffraction analysis. Furthermore, anti-proliferative investigations based on the derivatives of several cell lines for cancer show that the compounds have potential anti-cancer properties [2]. A promising anti-tumour drug, 9-(2′-hydroxyethylamino)-4-methyl-1-nitroacridine (C1748), was discovered to move through phase I metabolic pathways in a laboratory setting (Figure 2). The current research aimed to learn more about its metabolisation by phase II enzymes called UDP-glucuronosyltransferases (UGTs) as well as its potential for being involved in drug–drug interactions caused by UGT regulation [3].

Compound-induced cell-cycle arrest and cell death in human lung adenocarcinoma cells, Ataxia telangiectasia kinase, was initiated by all compounds, and histone H2A.X was phosphorylated at Ser139, indicating damage to DNA. The compounds enhanced the phosphorylation and accumulation of p53, which regulates the cell cycle and cell death [4].

In human colorectal HCT116 cells, oxidative stress plays a role in the anti-proliferative effect of acridine chalcone 1C ((2 E)-3-(acridin-9-yl)-1-(2,6-dimethoxyphenyl)prop-2-en-1-one) [5]. The anti-tumour effects of the new spiro-acridine (E)-50-oxo-10-((3,4,5-trimethoxybenzylidene)amino)-10,50-dihydro-10H-spiro[acridine-9,20-pyrrole]40-carbonitrile (AMTAC-17) were investigated. After an acute dose (2000 mg/kg, intraperitoneally, i.p.) in mice, the toxicity was assessed. The anti-tumour activity of AMTAC-17 (12.5, 25, or 50 mg/kg, i.p.) following seven days of treatment was studied using the Ehrlich ascites carcinoma model [6]. For 2-((6-chloro-2-methoxy-acridin-9-yl)amino)-5,6,7,8-tetrahydro-4H-cyclohepta [b], the toxicity and anti-tumour activity of -thiophene-3-carbonitrile (ACS03), a hybrid thiophene–acridine molecule with anti-leishmanial activity, was investigated. In vitro (on HaCat and peripheral blood mono nuclear cells) and in vivo (on zebra fish embryos and acute toxicity in mice) tests were performed. The anti-tumour activity of HCT-116 (human colon carcinoma cell line), K562 (chronic myeloid leukaemia cell line), HL-60 (human promyelocytic leukaemia cell line), HeLa (human cervical cancer cell line), and MCF-7 (breast cancer cell line) was also investigated in vitro and in vivo (Ehrlich ascites carcinoma model). HCT-116 cells were selectively inhibited by ACS03 (IC_50_ = 23.111.03 M, half-maximum inhibitory concentration) [7].

The acridine derivative dimethyl 2-[(acridin-9-yl) methylidene]-malonate (LPSF/IP-81) has optical characteristics. This molecule was attached to the ConcanavalinA (ConA) lectin and it is used as a sugar probe in lectin histochemistry according to the article. After conjugation, the hemagglutinating activity and LPSF/IP81 photoluminescence remained unchanged. The ConA structure was maintained via circular dichroism of ConA-LPSF/IP81conjugate. Normal, fibroadenoma, and invasive ductal carcinoma of the human breast were studied using Lectin histochemistry with the ConA-LPSF/IP81conjugate [8]. The novel synthetic lanthanum compound bis (acridine-9-carboxylate)-nitro-europium (III) dehydrate had anti-angiogenic and apoptotic effects in an animal model of carcinogenesis [9].

The anti-cancer drug 9-amino-1-nitroacridine (C-1748), developed in a laboratory setting, is a treatment option for pancreatic cancer. It involves the P450 3A4 isoenzyme and cytochrome P450 reductase (CPR), and the response’s modulation was investigated in the Panc-1, MiaPaCa-2, BxPC-3, and AsPC-1 cell lines of pancreatic cancer, which differed in terms of their expression levels and typically altered this cancer type’s genes. C-1748 had the strongest cytotoxic action against MiaPaCa-2 cells, but AsPC-1 cells, on the other hand, were the most resistant (IC_50_: 0.015, 0.075 M, respectively) [10]. Allosteric regulation of the a1Aand a1B-adrenergic receptors was demonstrated by a variety of 9-aminoacridine compounds. The 9-aminoacridines accelerate [3H] prazosin segregation from a1A- and a1B-adrenergic receptors and block receptor stimulation by the endogenous agonist norepinephrine in a non-competitive manner [11]. AT11-L0, which is generated from the AT11 DNA sequence, creates a single main parallel G-quadruplex (G4) conformation, and has anti-proliferative properties comparable to AT11 and AS1411 aptamers. Acridine orange derivatives, on the other hand, are a useful class of G4 ligands. Researchers tested AT11-L0 G4 as a supramolecular transporter for delivering acridine ligands C3, C5, and C8 to HeLa carcinoma cells in ref. [12].

The inhibitory activity of new acridine-based N-acyl-homoserine lactone (AHL) analogues has been examined in SAS (human oral squamous carcinoma cell line). At 5.3–10.6 M, one analogue caused G2/M phase arrest, while at a higher dose (21.2 M), it caused polyploidy [13]. A new class of acridine hydroxamic acid compounds was developed and produced as a potential new dual Topo and HDAC inhibitor. MTT studies revealed that each and every hybrid compound had substantial anti-proliferative effects with IC_50_ values in the low micromolar range, with compound 8c showing particularly strong action against U937 (IC50 = 0.90 M). It was also discovered that compound 8c had the best HDAC inhibitory action, being several times more effective than the HDAC inhibitor SAHA. At 50 micrograms, all of the substances inhibited Topo II according to subsequent tests. Furthermore, compound 8c had the potential to interact with DNA, resulting in apoptosis in U937 cells [14]. The authors designed and synthesised a unique small-molecule library with numerous modifications and replacements based on the CQ structure and an acridine skeleton, and then evaluated the molecules for efficient autophagy inhibition. They discovered that 9-chloro-2-(3-(dimethylamino) propyl) pyrrolo [2,3,4-kl] acridin-1(2H)-one (LS-1-10) was the most efficient inhibitor of autophagic-mediated degradation from their database, and that it could reduce the viability of various colon cancer cells [15]. DNA-intercalating agents are novel hetero-aromatic compounds with a cytotoxic moiety. Two iodinated acridine derivatives have been found to have a favourable in vivo kinetic profile for use in targeted radionuclide therapy. The goal of this research was to evaluate these drugs in a preclinical setting. Finally, an acridine derivative with increased nuclear localisation was shown to be a better option for ^125^I-targeted radionuclide therapy [16].

Based on the pharmacological characteristics of B16F0 tumours in mice, for melanoma-targeted 125I radionuclide therapy, two melanin-targeting radioligands were developed: [125I] ICF01035 and [125I] ICF01040. Researchers demonstrated in vitro that these compounds have different radiotoxicities in connection with melanin and the acidic vesicle contents in B16F0, B16F0 PTU, and A375 cell lines. ICF01040 was detected in the cytoplasmic vesicles of both types of melanomas, whereas ICF01035 was located in the nuclei of achromic (A375) and the melanosomes of melanised (B16F0) melanoma cells. In all cell lines, [^125^I] ICF01035 caused a similar survival fraction (A50) and a considerable drop in S-phase cells in amelanotic cell lines. In vivo, [^125^I] ICF01035 dramatically decreased the amount of B16F0 lung colonies, allowing the treated mice to live longer. Melanosomes or acidic vesicles could possibly be used to treat melanoma in the future [17]. An anti-cancer agent, 9-phenyl acridine (ACPH), was used. Normal cells, such as human lymphocytes and Chinese hamster V79 cells, were more susceptible to ACPH than A375 and HeLa, two human cancer cell lines. ACPH has been found to be a promising cancer chemotherapeutic agent. Through a mitochondria-mediated caspase-dependent route, ACPH administration caused cells to die apoptotically [18]. Nine different tetrahydroacridine derivatives containing an iodobenzoic moiety were produced and cancer cell lines were examined for cytotoxicity A549 (human lung adenocarcinoma), HT-29 (human colorectal adenocarcinoma), and EA.hy926 (human somatic cell line) (human umbilical vein cell line). All substances were more cytotoxic than the control agent etoposide, and 5-fluorouracil against A 549 (IC_50_ 59.12–14.87 M) and HT-29 (IC_50_ 17.32–5.90 M) cell lines [19].

The biological activities of 9-(2-(1-arylethylidene) hydrazinyl) acridine and its synthetic derivatives were designed, synthesised, and analysed. The free radical scavenging capacity of the produced compounds (**4a**–**4j**) was determined using a variety of biochemical tests. These chemicals were tested for anti-cancer efficacy in comparison with two human cancer cell lines, cervical cancer cells (HeLa) and liver cancer cells (HepG2), as well as a normal human embryonic kidney cell line (HEK 293) [20]. 9-phenylacridine (ACPH), an acridine derivative, was discovered to have anti-cancer action in both cell lines and an in vivo model. Researchers used photo-cleavage experiment to look at the effects of ACPH on in vitro DNA before UVA exposure. In cultivated A375 melanoma cells, the effect of such treatment was also investigated. ACPH could sensitise UVA-induced DNA damage in vitro and in cells, according to their findings [21]. A family of acridine derivatives as effective and selective inhibitors of the IRE1-XBP1 branch of the UPR with substantial cytotoxicity on MM cells as well as in vivo MM tumour growth using TDA analysis on HTS was discovered [22]. N0-(2-chloro-6-methoxy-acridin-9-yl)-2-cyano-3-(4-dimethylaminophenyl)-acrilohidrazida was synthesised and its toxicity and anti-cancer efficacy were assessed (ACS-AZ10). In vivo, ACS-AZ10 shows a significant anti-cancer effect and is quite safe [23].

EGFR and PKCs are inhibited by acridine yellow G, as a result of which cell development is inhibited, cell-cycle arrest occurs in the G1phase, brain tumours shrink. Acridine yellow G has IC_50_ values of 7.5 and 5 M for EGFR and PKCs, respectively [24]. New promising unsymmetrical bisacridine derivatives (UAs) have been created. The condensation of 4-nitro or 4-methylacridinone, imidazoacridinone, and triazoloacridinone derivatives with 1-nitroacridine molecules connected with an aminoalkyl chain yielded three groups of 36 compounds. The great effectiveness of these compounds against many tumour cell lines was discovered by cytotoxicity testing [25].

A one-pot four-component cyclo-condensation of dimedone for the synthesis of 9-aryl-hexahydro-acridine-1,8-diones that is easy, efficient, and cost-effective against the HepG2 and MCF-7 cell lines, and some of the acridine-diones synthesised were discovered to have promising anti-cancer activity [26]. Four mesothelioma cell lines to assess the anti-proliferative effect of a variety of acridine-based catalytic inhibitors of hTopo II (H513, H2372, H2461, and H2596) were applied. The results showed that these compounds inhibited malignant cell proliferation with EC50 values ranging from 6.9 to 32 M. The Guava Nexin assay and PARP cleavage data showed that apoptosis is the primary reason for cell apoptosis. The results support earlier research on pancreatic cancer and hTopo II catalytic inhibitors, suggesting that substituted acridines may be effective in the treatment of malignant mesothelioma [27].

The synthesis and pharmacological assessment of a set of bifunctional acridine-HSP90 (Cpd: 2.1.23) inhibitor ligands as telomerase inhibitors were reported. Using a click-chemistry technique, four hybrid acridine-HSP90 inhibitor conjugates were created and demonstrated to have comparable effects in the TRAP-LIG telomerase test to the well-known telomerase inhibitor BRACO-19. The conjugates also showed significant cytotoxity in the sub-M range against a variety of cancer cell lines [28]. The interaction of three novel diphenyl-substituted spiro triazolidine- and thiazolidinone-acridines (Cpd: 2.1.24) with calf thymus DNA was studied using UV-vis, fluorescence, circular dichroism spectroscopy, and viscometry [29].

Early investigations were conducted on the amino acid-attached acridines as potential anti-cancer medication leads. The chemicals have substantial anti-proliferative effect, as evidenced by the MTT assay, phase contrast micrographs, and Confocal pictures of immune-labelled C6 Glioma cells for markers such as a-tubulin, GFAP, mortalin, and HSP-70 cells. Flow cytometry data revealed that the chemicals also stopped cells in the G0/G1 phase of the cell cycle [30]. A novel family of tri-substituted acridines that would imitate the actions of BRACO19 was described. These compounds were made by adding heteroacyclic moieties to the BRACO19 molecular structure at positions 3 and 6. The human telomeric DNA quadruplex was stabilised by all of the studied derivatives. The novel derivatives were all capable of folding single-stranded DNA sequences into anti-parallel G-quadruplex structures according to the findings. When compared with the HT 29 cancer cell line, the studied compounds were less harmful to human fibroblast cells [31].

**Figure 3:**
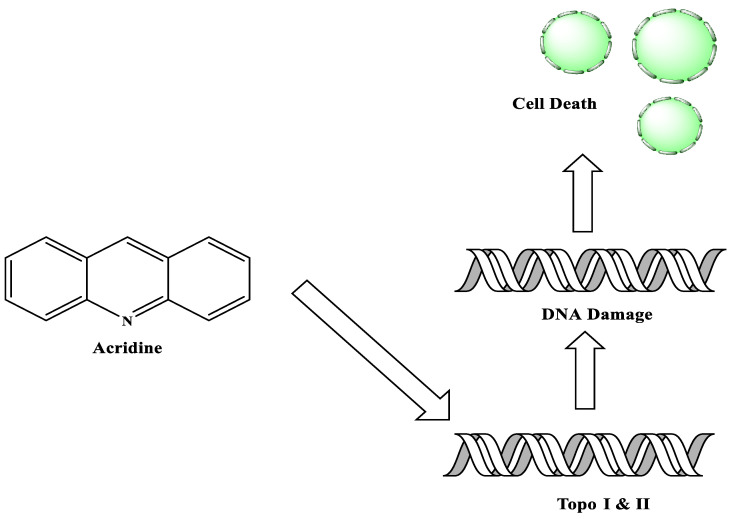
Schematic diagram of acridine as an inhibitor of topoisomerase I and II.

When a non-covalent contact such as intercalation is used, the synthesis of DNA–polymer hybrids is simple. The exact structure and characteristics of the polymer play a significant role in the strength of the connection. The production of discrete, well-defined nanoparticles can be carried out by simply combing the components together in an aqueous solution and utilising a DNA sequence of specific length [32]. CuGGHK-Acr, a novel DNA-cleaving agent that uses a catalytic metallo drug to target G4 telomeric DNA, was reported. CuGGHK-Acr can selectively bind to G4 telomeric DNA in comparison with CT-DNA and facilitate an effective irreversible cleavage of G4 telomeric DNA in comparison with telomeric DNA in other structural states, according to these findings [33]. The anti-proliferative properties of a series of 9-benzyl acridine derivatives were investigated. At 100 M, each and every compound mentioned had substantial Topo II inhibitory action. Through a caspase-dependent intrinsic mechanism, the usual chemical 8p demonstrated significant DNA-binding capacity, generated DNA double-strand breaks, and promoted death in A549 cells (Table 1 and Figure 3) [34].

### 2.2. 9-Amino Acridine

Four NSCLC cell lines were used to test the anti-proliferative effect of a range of acridine-based catalytic inhibitors of TOPOII (H460, A549, H2009, and H2030) [35]. The current findings in the treatment of breast cancer showed that CK0403 was more potent and effective than CK0402 against estrogen receptor-negative and HER2-overexpressing breast cancer cell lines, implying that it could be used as a breast cancer chemotherapy in the future [36].

Interactions between specific distinct moieties of 9-amino acridines and DNA were investigated and demonstrated to be important in determining the overall stabilities of DNA G-quadruplex complexes. Both 9-amino acridines were found to produce varying levels of structural stability through intercalation, although having equal binding affinities to the G-quadruplex. This distinctive trait of modifying structural stability is most likely a factor in influencing telomerase function and, as a result, the reported anti-cancer activity varied between the two 9-amino acridines [37].

**Figure 4:**
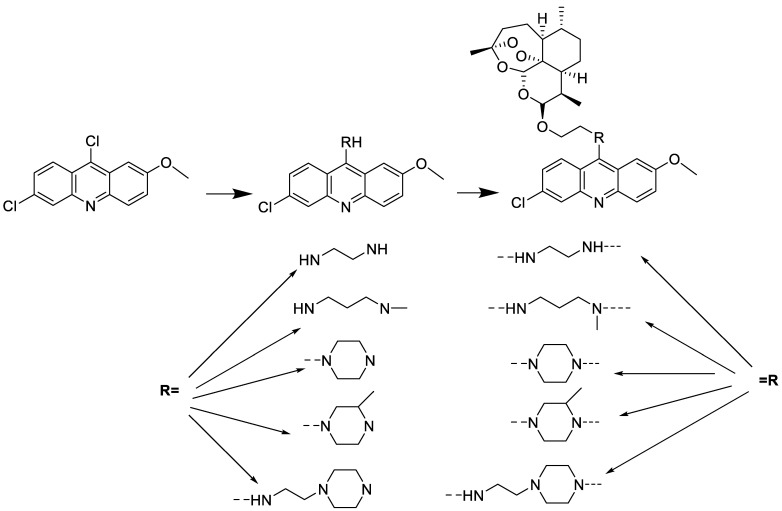
Multi-step synthesis of artemisinin–acridine hybrids and their derivatives.

The anti-malarial activity of 9-aminoacridine and artemisinin–acridine hybrid compounds against both the chloroquine sensitive but also gametocytocidal strain (NF54) and the chloroquine resistant (Dd2) *Plasmodium falciparum* strains was determined in vitro. CHO cell cytotoxicity, HepG2 and SH-SY5Y apoptosis, and anti-cancer efficacy against HeLa cell lines were all tested in vitro (Figure 4) [38]. 9-aminoacridine (9AA) showed specific toxicity for infectious leukemic cells regardless of their p53 status due to p53 reactivation and NF-B inhibition. It was also shown that 9AA stimulates caspase-3/7, which results in PARP cleavage. The effectiveness of 9AA in the MET-1 ATL model was also studied [39].

The 9-aminoacridine carboxamide Pt complexes (Cpd: 2.2.5) have been reported to bind at 50-CpG sequences and have the effect of CpG methylation on cisplatin-analogue DNA binding. Using cisplatin, the results of their binding to methylated and unmethylated 50-CpG sequences were compared [40].

An identified series of 21 compounds of 9-aminoacridine derivatives with an acridine scaffold were synthesised and examined for their anti-proliferative activity against K562, HepG-2, and MCF-7 cells as potentially interesting novel dual VEGFR-2 and Src inhibitors [41]. Four acridine Pt complexes’ specificity in DNA sequences was evaluated and compared to that of cisplatin [42]. A small library of 9-aminoacridine derivatives that were substituted with topoII catalytic inhibitory characteristics was identified. In this study, the capacity of the compounds and derivatives to decrease proliferation and trigger cellular apoptosis in SCLC was investigated (Table 2) [43].

### 2.3. 9-Anilino Acridines

Nucleophilic substitution of 2-methyl-9-chloroacridine (AS) with aromatic amines yielded numerous 2-methyl-9 substituted (AS 0–8) acridines. The MTT assay was used to test three substances for anti-proliferative activity against A-549 (human small-cell lung carcinoma) and MCF-7 (human breast cancer) cell lines. The cytotoxicity of acridines against cancer cells was shown to be more active in the A-549 cell line than in the MCF-7 cell line [44]. The synthesis of molecular hybrids with high efficiency with a 9-anilinoacridine (9-AnA) core that intercalates DNA and a methyl triazene DNA-methylating moiety has been reported [45]. The anti-tumour activity of the chimeras was tested. In anti-proliferative experiments with multiple cancer cell lines, Chimera 7b showed the best anti-cancer activity at low micromolar IC_50_ values [45].

**Figure 5:**
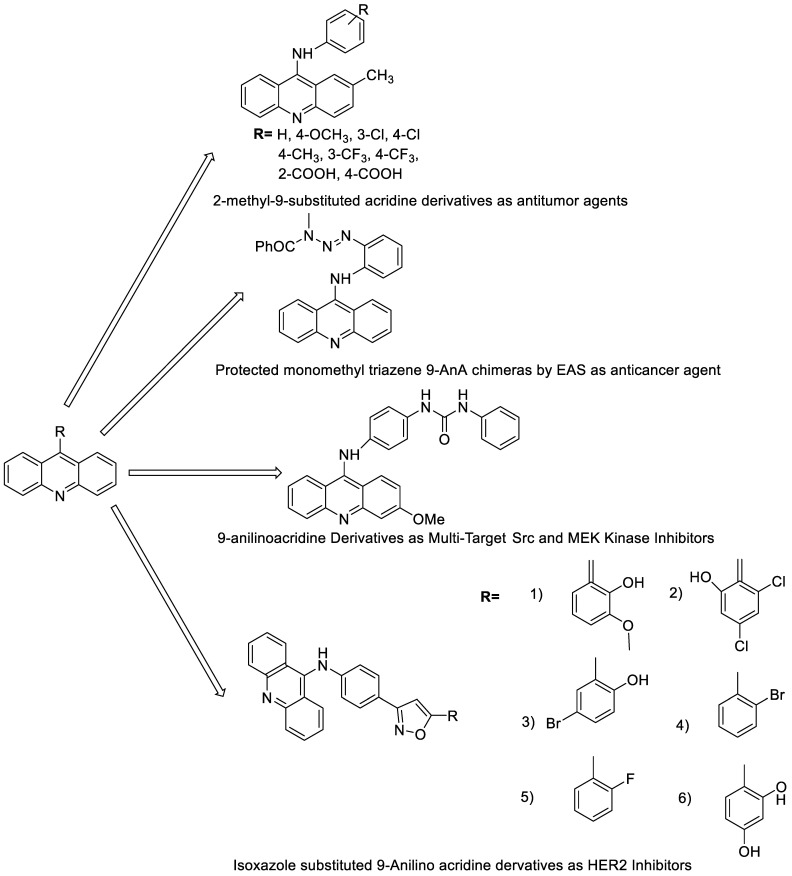
9-anilino acridine derivatives showing antitumour activity.

Researchers have developed and synthesised a new series of 9-anilinoacridines incorporating phenyl-urea moieties as possible new dual Src and MEK inhibitors. In vitro anti-proliferative studies on K562 and HepG-2 tumour cells revealed that the majority of the compounds were cytotoxic. According to their findings, the acridine scaffold, notably compound 8m, could be helpful in the creation of novel multi-target Src and MEK kinase inhibitors [46]. BO-1051 decreased cell viability in oral cancer cells with a low IC_50_, but not in normal gingival fibroblasts. BO-1051-induced tumour suppression was followed by cell-cycle arrest and downregulation of stemness genes according to cell cycle analyses. It was shown that BO-1051 had cytotoxic activity by causing the induction of autophagy and cell-cycle arrest. It was proposed that combining BO-1051 with radiation could be a viable option for oral cancer in the future [47]. In silico designs were used to create several novel isoxazole substituted 9–anilinoacridines (1a–z) with HER2 inhibitory activity. Using Schrodinger suit 2016-2, docking studies of compounds 1a–z were conducted on HER2 (PDB id-3PP0). This work adds to the evidence that isoxazole substituted 9-aminoacridine compounds could be used as HER2 inhibitors (Table 3 and Figure 5) [48].

### 2.4. Acridine Thiourea Gold

Two novel 1-acridin-9-yl-3-methylthiourea Au(I) DNA intercalators have been developed: [Au (ACRTU)2]Cl (2) and [Au(ACRTU)(PPh3)]PF6. Both complexes were extremely active in the cisplatin-sensitive A2780 human ovarian cancer cell line, with IC_50_ values in the sub-micromolar range. MDA-MB-231 (triple negative), SK-BR-3 (HER2+, ERα-, and ERβ-), and MCF-7 (ER+) are all cytotoxic to different phenotypes of breast cancer cell lines [49]. The synthesis of seven new cyclometalated Au(III) complexes, five of which contain an acridine moiety linked via (NO) or (NN) chelates, acyclic amino carbenes (AAC), and N-heterocyclic carbenes (NHC), was reported [50]. The anti-proliferative properties of the various complexes were investigated in vitro on a panel of cancer cells, including leukaemia, lung, and breast cancer cells. In some of the series representative substances, researchers observed a relationship between cytotoxicity and intracellular gold uptake. Some of the acridine-decorated compounds were shown to interact with ds-DNA using FRET-melting techniques (Table 4) [50].

### 2.5. Acridine-Thiazolidinone

The interactions of three novel acridine–thiazolidinone compounds (**2a**–**2c**) using calf thymus DNA and a variety of cell lines (leukaemic cells HL-60 and L1210, and human epithelial ovarian cancer cell lines A2780) were investigated. Compounds **2a**–**2c** had a high affinity for calf thymus DNA, with regard to binding constants ranging from 1.37 ×10^−6^ to 5.89 × 106 M_1 as determined by spectrofluorimetry. After 72 h of incubation, all of the investigated compounds showed substantial cytotoxic activity in vitro, with IC_50_ values of 1.3 ± 0.2 M (HL-60), 3.1 ± 0.4 M (L1210), and 7.7 ± 0.5 M (A2780). Acridine compounds were rapidly accumulated by cancer cells, and alterations in glutathione levels were confirmed. Cell proliferation was suppressed by the chemicals and resulted in cell-cycle arrest and cell death. Their ability to have an effect on cells was connected to thiol reactivity and DNA-binding activity [51]. N-alkylation and the Michael reaction were used to create a series of unique hybrid 5-acridin-9-ylmethylene-3-benzyl-thiazolidine-2,4-diones. A 3-(4,5-dimethylthiazol-2-yl)-2,5-diphenyltetrazolium bromide (MTT) assay was employed to assess cell viability, and DNA interaction experiments were carried out using electrochemical methods (Table 5) [52].

### 2.6. Acridinone

A class of acridinones derived from the structure of podophyllo toxin were discovered as a result of a lead discovery effort aimed at less structurally complex synthetic chemicals. Wound-healing experiments using the metastatic and triple-negative breast cancer cell line MDA-MB-231 were used to test the drugs in vitro. Four compounds were discovered with IC_50_ values ranging from 0.294 to 1.7 μM [53]. The cytotoxic activity of a new series of 9 (10H)-acridinone-1,2,3 triazole derivatives against human breast cancer cell lines was developed, synthesised, and assessed, 2-methoxy-10-((1-(4-methoxybenzyl). The most potent compound against MCF7 cells was 1H-1,2,3-triazol-4-yl)methyl)acridin-9(10H)-one 8c (IC_50_ = 11.0 ± 4.8 µM), which was more potent than toposide (IC_50_ = 12.4 ± 4.7 µM) [54]. The designed UGT1A10’s capacity and selectivity in the glucuronidation of acridinone anticancer drugs in a cellular setting was tested. These results imply that extrahepatic UGT1A10 is involved in the metabolism and bioactivation of C-1305, and they provide a foundation for more mechanistic research into the drug’s mode of action. A translational study into the involvement of this enzyme in the regulation of C-1305 toxicity in cancer was also conducted (Table 6) [55].

### 2.7. Benzimidazole Substituted Acridines

**Figure 6:**
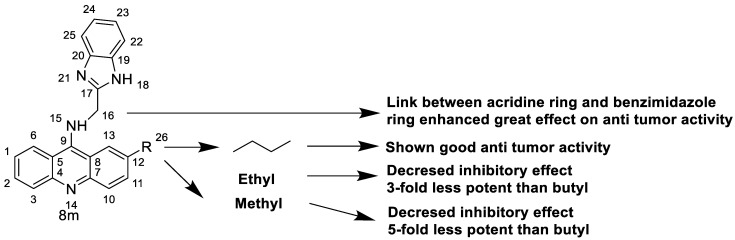
SAR of acridine benzimidazole.

In human colon cancer cell lines, a novel benzimidazole acridine derivative (8m) demonstrated cytotoxic action. This derivative, 8m, activated both the intrinsic and extrinsic death pathways in a time- and concentration-dependent way according to the study. Further research into the ROS-JNK1 pathway’s mode of action revealed that it was critical in initiating 8m-induced apoptosis. Researchers also showed that 8m can upregulate DR5, which is characteristic for potent anticancer drugs [56]. Dual Topo and PARP-1 inhibitors, a series of 4-amidobenzimidazole acridines, were designed and synthesised. Compound 11l had powerful inhibiting effects on Topo and PARP-1 as well as a considerable inhibitory effect on cancer cell proliferation. According to their findings, single drugs that inhibit Topo and PARP simultaneously could be used as an alternative to cancer treatment, and 11l could be a possible lead chemical for anticancer drug discovery [57]. A series of new DNA-targeted agents (Figure 6), unique benzimidazole derivatives of acridine, were designed and synthesised (Table 7) [58].

### 2.8. Benzoacridine

Potential anti-cancer drugs and tubulin polymerisation inhibitors were designed and synthesised. The anti-cancer activity of the synthesised compounds was assessed using the 3-[4,5-dimethylthiazol-2-yl]-2,5-diphenyl tetrazolium bromide (MTT) assay against eight cancer cell lines, including MCF7, A2780, HeLa, HepG2, DU145, A549, PC3, and LNCAP cancer cells, as well as normal human umbilical vein endothelial cells (HUVEC), with lapachone. With IC_50_ values ranging from 5.23 to 24.32 μM, some of the compounds (**4c** and **4g**) displayed substantial cytotoxic effects on cancer cells [59]. Novel 7-substituted-5, 6-dihydrobenzo[c]acridine derivatives were designed and synthesised. According to recent biophysical research, the substitutes may effectively attach to and stabilise the c-KIT G-quadruplex with significant selectivity towards duplex DNA. The biological examination of compound 2b revealed that it might induce apoptosis by activating the caspase-3 cascade pathway [60].

A rapid one-pot microwave-assisted synthesis of novel deadly phenanthrene fused-tetra hydrodibenzo-acridinones was reported [61]. This protocol provides a broad substrate range, catalyst-free synthesis, atom-economy, simple recrystallisation, good yields, and the use of ethanol as a green solvent. The in vitro cytotoxicity of these novel compounds was tested against cervical (HeLa), prostate (PC-3), fibrosarcoma (HT-1080), and ovarian (SKOV-3) cancer cells, and they were found to be safer than the normal (Hek-293T) kidney cell line [61]. The development of a 12-arylbenzoacridine library using an approach centered on diversity resulted in non-toxic estrogenic and anti-estrogenic compounds. The estrogen receptors (ER alpha) and ER beta (IC_50_ lM) have a strong binding affinity for derivatives with a hydroxy group at the molecular edge, but binding to the estrogen-related receptor c (ERRc), an orphan nuclear receptor on which estrogens frequently trigger unfavourable events, was not observed. According to the findings, 12-arylbenzoacridines can be used as a new platform for the creation of selective estrogen-receptor modulators (SERMs) (Table 8) [62].

### 2.9. Imidazoacridinone

**Figure 7:**
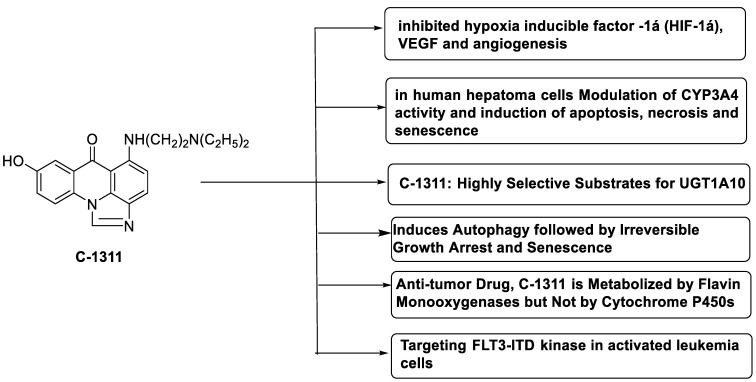
Schematic diagram of C-1311 and its activity.

C-1311 is an anti-tumour imidazoacridinone that inhibits DNA-reactive topoisomerase II and the FLT3 receptor tyrosine kinase. Researchers showed that C-1311 inhibits new targets such as hypoxia-inducible factor-1α (hIF1), vascular-endothelial growth factor (VeGF), and angiogenesis in this study. C-1311 is a potent inhibitor of hIF-1 α, VeGF, and angiogenesis, according to the findings, which offer fresh insights into the mechanism underlying its anti-cancer activity [63]. Photo-rupture of IA-loaded lysosomes and tumour cell lysis via production of reactive oxygen species were used in a novel photo activation-based pharmacological Trojan horse method to target and destroy MDR cancer cells. It was discovered that MDR cells’ Achilles heel is lysosomal sequestration of IAs, which may be exploited to destroy MDR tumour cells by lysosomal photo death [64]. In hepatoma cells, the effect of CYP3A4 overexpression on the cellular response was generated by the anti-tumour drug C-1311. The effect of CYP3A4 overexpression on C-1311 metabolism and the modification of CYP3A4 activity by C-1311 were also investigated. It was stated that when evaluating the possible therapeutic effects of C-1311, inter-patient variability in CYP3A4 levels should be taken into account (Figure 7) [65].

A credible QSAR was constructed using computational molecular modelling, and the linking potency of inhibition was used to calculate binding affinity. In addition, the crystal structures of NQO2 with two imidazoacridin-6-ones was solved. Finally, one of the N-oxides inhibited the enzymatic action of NQO2 in cells, suggesting that it might be used as a pharmacological probe to explore the enzyme’s characteristics in vitro and in vivo [66]. UDP-glucuronosyltransferases (UGTs) are human enzymes that can glucuronidate these two molecules. The activities of human recombinant UGT1A and UGT2B isoforms and microsomes from the human liver (human liver microsomes (HLM)), the entire human intestinal mucosa (human intestinal microsomes (HIM)), and seven isolated segments of the human gastrointestinal tract were investigated using high-performance liquid chromatography. It was discovered that in vivo glucuronidation of imidazoacridinone and triazoloacridinone medications occurs in the human liver and gut, which may open the door for future translational research into the role of UGTs in drug resistance [67].

A549 and H460, human non-small-cell lung cancer (NSCLC) cell lines, were used to study the distinct sequence of cellular responses to C-1311. C-1311 (IC_80_ 5 0.08 mM) triggered G1 and G2/Marrests in A549 cells, although H460 cells (IC_80_ 5 0.051 mM) accumulated primarily in G1. According to the studies, autophagy induction precedes and confirms a C-1311-induced senescence pathway in NSCLC, although it is not the sole determinant [68]. That function of certain liver enzymes affects the metabolism of C-1311 and its less-active 8-methyl derivative, 5-diethylaminoethylamino-8-methoxyimidazoacridinone (C-1330). C-1311 and C-1330 were digested by live human microsomal enzymes but not by any of the human recombinant cytochromes P450 enzymes examined (P450s). Both drugs inhibited two of these enzymes, CYP1A2 and CYP3A4 [69]. The DNA-reactive inhibitor of topoisomerase II imidazoacridinone C-1311 has previously been proven to be a strong and selective inhibitor of recombinant FLT3. Researchers investigated the effect on leukaemia cells with wild-type FLT3, FLT3-ITD mutant, and no FLT3 receptor to add to their findings. It was reported that more preclinical and clinical research into its potency against the FLT3-ITD kinase is warranted (Table 9) [70].

### 2.10. Nitroacridine

**Figure 8:**
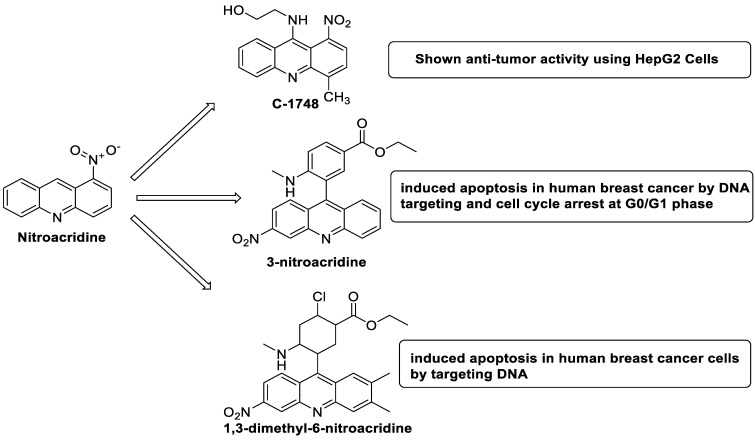
Mechanism of nitroacridine targeting various pathways.

The differential in toxicity between C-1748, 9-(20-hydroxyethylamino)-4-methyl-1-nitroacridine, and its 4-demethyl counterpart, C-857, was shown to be due to changes in metabolic routes for the two chemicals; the effect of decreasing and/or hypoxic circumstances on metabolism was also examined. Finally, the importance of hypoxic circumstances and POR’s direct involvement in the metabolism of both substances was established. C-1748’s low reactivity and the stability of its metabolites, compared with C-857, are thought to have a substantial role in the compound’s reduced toxicity in animals [71]. DNA damage is caused by interactions with DNA and interference with regulatory mechanisms. The structure–activity connection revealed that substituents on position 9 of 3-nitroacridine derivatives were crucial for DNA-binding and anti-proliferative effects. Compounds **1**, **2**, and **3** were reported to have a high affinity for DNA and a favourable inhibitory effect on tumour cell proliferation, and could be established as viable candidates for additional chemical optimisation [72]. By changing the substituted group on position 9 of 1,3-dimethyl-6-nitroacridine, a new series of 1,3-dimethyl-6-nitroacridine derivatives were synthesised. Researchers tested four cancer cells for antitumour efficacy. They stopped breast cancer cells from growing by inducing apoptosis by targeting DNA and stopping cell-cycle progression in the G2/M phage. The structure–activity connection suggested that 9-amines were crucial in DNA-binding and anti-proliferative properties (Table 10 and Figure 8) [73].

### 2.11. Oxazine

**Figure 9:**
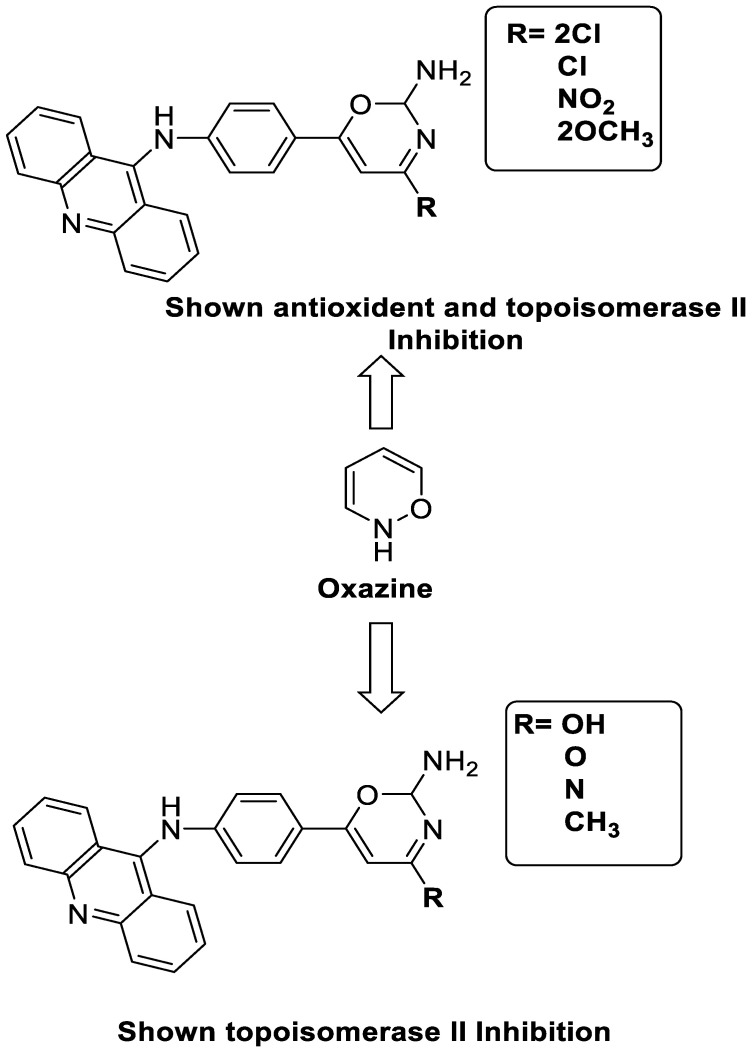
Oxazine as an antioxidant and topoisomerase II inhibitor.

A series of 9-anilinoacridines substituted with oxazine derivatives were synthesised and tested in vitro for antioxidant and anti-cancer activity against Daltons Lymphoma Ascites (DLA) cell growth. These compounds were found to have significant antioxidant and anti-cancer activity (inhibition of DLA cell proliferation). Compounds **5a**, **5h**, **5i**, and **5j** were the most cytotoxic, with CTC_50_ values ranging from 140 to 250 mg/mL. Using the Schrodinger Maestro 9.2 version, the docking studies of the synthesised derivatives were performed towards the key Topoisomerase II (1QZR). The oxazine-substituted 9-anilinoacridine derivatives **5a**, **5h**, **5i**, and **5j** have been reported to have significant anti-cancer activity as topoisomerase II inhibitors [74]. Docking investigations against topoisomerase II were conducted using new oxazine-substituted 9-anilinoacridines. Compounds **1c**, **1f**, and **1g** had a high Glide rating. In addition, in silico ADMET screening was carried out [75]. Series of oxazine-substituted 9-anilinoacridines were synthesized, characterized, and tested for anti-cancer efficacy against Dalton’s lymphoma ascites cells utilising in vitro and in vivo methodologies. On Dalton’s lymphoma ascites cells, these conjugates showed strong antitumour activity according to the findings. Compounds **4b**, **4c**, **4e**, and **4j** were the most cytotoxic, with CTC_50_ values ranging from 96.5 to 190 g/mL (0.125 to 0.352 µM). The PHASE module of the Schrodinger suite was used to conduct 3D QSAR research (Table 11 and Figure 9) [76].

### 2.12. Platinum-Acridine Anti-Cancer Agents

A validated genome-wide screening approach was used to examine the spectrum of activity of a library of non-classical platinum-acridine hybrid substances in *Saccharomyces cerevisiae* and the distantly related yeast Schizo saccharomyces pombe. Chemo-genomic profiles of *S. cerevisiae* and *S. pombe* revealed that many platinum-acridines cause DNA damage that differs from cisplatin even though they require different DNA-repair modules [77]. Carbamate-coupling chemistry was used to synthesise a new pharmacophore containing a DNA-targeted platinum–acridine hybrid agent and estrogen receptor-targeted 4-hydroxy-N-desmethyl tamoxifen (endoxifen) and perform its analysis in breast cancer cell lines [78].

MWCNTs coated with a non-classical platinum chemotherapeutic agent ([PtCl(NH3)2(L)] Cl (P3A1; L = N-(2-(acridin-9-ylamino)ethyl)-N-methylproprionimidamide) and 1,2-distearoyl-sn-glycero-3-phosphoethanolamine-N-[amino(sn-glycero-3-phosphoethanolamine-N-[amino(sn-glycero-3-phosphoethanolamine-N-[amino(sn-g (DSPE-mPEG) were reported. This finding suggests that employing MWCNTs as a medication carrier to deliver P3A1 to cancer cells could be advantageous for cancer chemotherapy and photothermal therapy [79].

The configuration of unique seven-membered, sterically overloaded chelates [Pt(en)(L/L0)](NO3)2 (4a/4b) from effective hybrid anti-tumour compounds [PtCl(en)(LH/L0H)](NO3)2 (**3a**/**3b**), where en is ethane-1,2-diamine and L(H) and L0(H) are (protonated) N-(2-(Compounds **3a** and **3b** have IC_50_ values of 12 (2 and 2.8(0.3 nM, respectively)))), and inhibit H460 lung cancer cell proliferation [80]. Carboxylic acid ester-group-containing platinum–acridine hybrid agents (Cpd: 2.12.5) were created. In the cell lines for ovarian cancer (OVCAR-3) and breast cancer (MCF-7, MDA-MB231), the most effective derivatives and parent chemicals that had not been modified were demonstrated as having up to 6-fold greater action than cisplatin. Cell proliferation was inhibited 80- and 150-fold at nanomolar doses in pancreatic (PANC1) and non-small-cell lung (NSCLC, NCI-H460) cancer cells, respectively [80].

A platinum–acridine hybrid agent [PtCl(en)(L)](NO3)2 (complex 1, en = ethane-1,2-diamine, L = 1-[2-(acridin-9-ylamino)ethyl]-1,3-dimethylthiourea was studied using a combination of biophysical, biochemical, and computational techniques to determine mechanistic distinctions between it and a much more effective second-generation analogue. N-methylpropionamidine is a specific type of N-methylpropionamidine [81]. Five NSCLC cell lines were resynthesised and assessed, indicating big cell, squamous cell, and adenocarcinomas. 7-Aminobenz[c] acridine was identified as a promising scaffold in a hybrid drug (P1–B1) and showed 32-fold enhanced tolerability in mice compared with the parent platinum–acridine (P1–A1) while maintaining sub-micromolar activity in numerous DNA-repair-proficient and p53-mutant cancer models [82].

**Figure 10:**
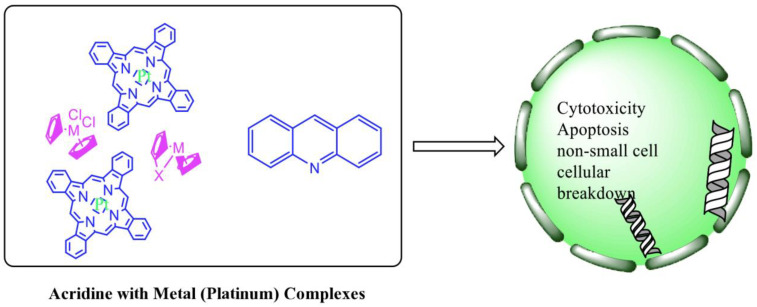
Schematic diagram of acridines with metal (platinum) complexes.

Binds generated by the platinum–acridine agent [Pt Cl(en)(N-(2-(acridin-9-ylamino)ethyl)-N-methylpropionimidamide)] were structurally characterised using high-performance liquid chromatography (HPLC) in combination with electrospray mass spectrometry (LC-ESMS). In cell-free DNA, (NO3)2 (compound **1**) was present [83]. [PtCl(en)(LH)], a platinum-acridine anti-cancer agent, [en = ethane-1,2-diamine, LH = N-(2-(acridin-9-ylamino)ethyl)-N-methylpropion imidamide], an acridinium cation, and (NO3)2 (1) [en = ethane-1,2-diamine, LH = N-(2-(acridin-9-ylamino)ethyl)-N-methylpropion imidamide], acridinium, were studied. The cytotoxic potency and cell-kill mechanisms of and the therapeutic medication cisplatin were investigated in chemo-resistant cell lines such as non-small-cell lung cancer (NSCLC). Compound **1** revealed a 40200-fold cytotoxic increase compared with cisplatin in the three studied cell lines (NCI-H460, NCI-H522, and NCI-H1435) at inhibitory doses approaching the low-nano molar range (Table 12 and Figure 10) [84].

### 2.13. Quinacrine

**Figure 11:**
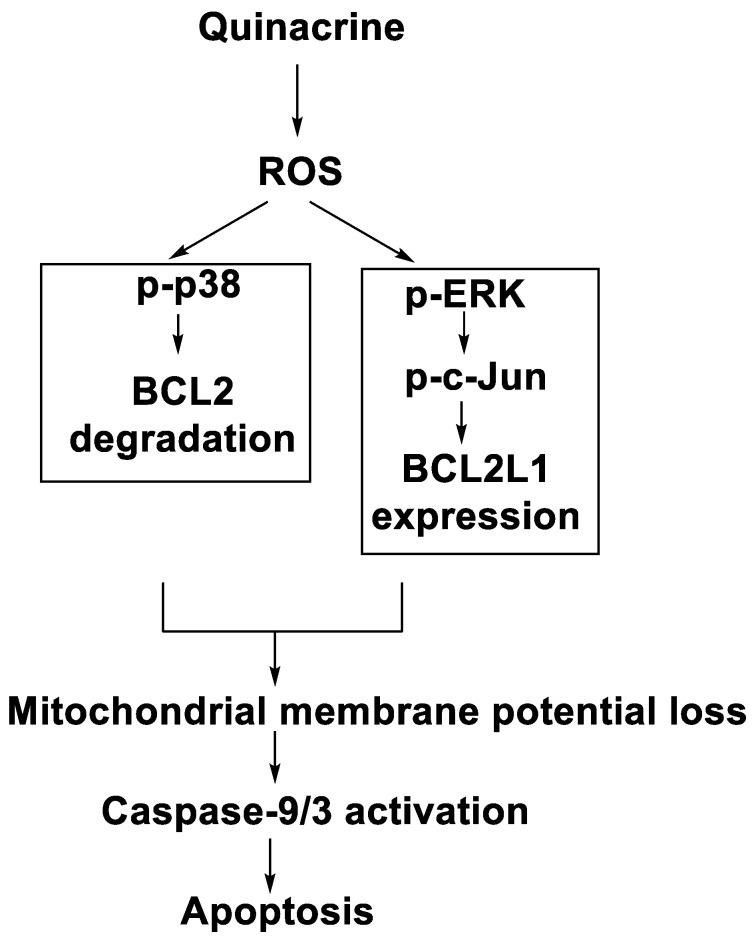
Signalling pathway responsible for the qunacrine-induced apoptotic death of K562 cells.

In human leukaemia K562 cells, the cytotoxic effect and mechanism of quinacrine activity were investigated. Quinacrine promoted apoptosis in K562 cells as well as ROS production, mitochondrial depolarisation, and BCL2L1 and BCL2 down-regulation. Quinacrine-induced cell death in K562 cells, according to the results, was mediated by mitochondrial changes caused by p38 MAPK-mediated BCL2 down-regulation and suppression of ERK/c-Jun-mediated BCL2L1 expression (Figure 11) [85].

Quinacrine’s cytotoxic effect on human leukaemia U937 cells was investigated. Quinacrine-induced apoptosis in U937 cells was followed by the production of reactive oxygen species (ROS), mitochondrial depolarisation, and the upregulation of BAX. Quinacrine-treated U937 cells displayed ROS-mediated p38 MAPK activation and ERK inactivation, which increased FOXP3 transcription [86]. Curcumin (Cur) and quinacrine (QC) were tested in vitro for their anti-proliferative efficacy against CSCs. Cur and QC reduced the proliferation, migration, and invasion of CSCs enriched side population (SP) cells generated by cigarette smoke condensate and induced breast epithelial transformed (MCF-10A-Tr) metastatic cells in a synergistic manner. The findings showed that combining Cur and QC promotes CSC mortality by raising the concentration of QC in the cells, inducing DNA damage and blocking DNA-repair pathways through regulating ABCG2 activity [87]. The findings provide systematic experimental evidence for APC’s role in ABT-888-mediated suppression of PARP-1PARylation, which leads to BER downregulation in response to QC-induced DNA damage [88]. The goal of this investigation was to see if QC has any cytotoxic effects on DLBCL cells. In the DLBCL cell lines SU-DHL-8 and OCI-LY01, QC triggered G0/G1 cell-cycle arrest and apoptosis and inhibited the production of the Myc proto-oncogene protein in a dose-dependent manner. According to the findings of this investigation, QC could be a promising anti-DLBCL medication [89]. Combining the 9-aminoacridine scaffold and the [1,3] thiazinan-4-ones group, 23 novel quinacrine (QC) derivatives were designed, synthesised, and tested. The majority of these hybrids demonstrated potent anti-cancer properties [90]. As an effort to develop effective and selective anti-cancer agents, the researchers designed, synthesised, and tested 23 novel quinacrine derivatives derived from the hybridisation of the quinacrine core scaffold and thiazolidin-4-ones (Table 13) [91].

### 2.14. Thiazacridine

Three novel thiazacridine compounds were synthesised and their anti-proliferative activities were evaluated. Three new thiazacridine compounds were produced and described using a three-step synthetic reaction: (Z)-5-acridin-9-ylmethylene-3-(4-methyl-benzyl)-4-thioxo-thiazolidin-2-one (LPSF/AC-99), (Z)-5-acridin-9-ylmethylene-3-(4-chloro-benzyl)-4-thioxo-thiazolidin-2-one (LPSF/AC-119), and (Z)-5-acridin-9-ylmethylene-3-. Colorimetric assays were used to test toxicity and selectivity. According to the findings, none of the chemicals were toxic to normal human cells and caused neoplastic cell death primarily through apoptosis [92]. A novel cytotoxic drug with acridine and thiazolidine nuclei the cytotoxic activity of four ATZDs was examined in human colon cancer HCT-8 cells: (5Z)-5-acridin-9-ylmethylene-3-(4-methylbenzyl)-thiazolidine-2,4-dione-AC-4; (5ZE)-5-acridin-9-ylmethylene-3-(4-methylbenzyl)-thiazolidine-2,4-dione-AC-4 -3-(4-bromobenzyl)thiazolidine-2,4-dione-AC-7;(5Z) -5-(acridin-9-ylmethylene) -3-(4-chlorobenzyl) -3-(4-chlorobenzyl)-1,3-thiazolidine-2,4-dione-AC-10; and -1,3-thiazolidine-2,4-dione-AC-10 (5ZE) AC-23 is a -5-(acridin-9-ylmethylene)-3-(4-fluoro-benzyl)-1,3-thiazolidine-2,4-dione. All of the ATZDs tested inhibited HCT-8 cell proliferation in a concentration- and time-dependent manner. According to the findings, ATZD inhibited the activity of DNA Topo isomerase I and affected tumour cell apoptosis via apoptotic pathways [93]. A number of novel thiazacridine compounds have been produced and tested as anti-cancer agents, both in terms of cytotoxicity and selectivity. All compounds had cytotoxic activity and selectivity according to the cytotoxicity assay. The most promising molecule was 3-acridin-9-ylmethyl-5-(5-bromo-1H-indol-3ylmethylene)-thiazolidine-2,4-dione (LPSF/AA29—**7a**), with IC_50_ values ranging from 0.25 to 68.03 mM depending on cell lineage. In HepG2 cells, the IC_50_ value for -thiazolidine-2,4-dione (LPSF/AA36—**7b**; 46.95 mM) was the lowest. None of the produced compounds were shown to be harmful to normal cells (IC_50_ > 100 mM) [94].

**Figure 12:**
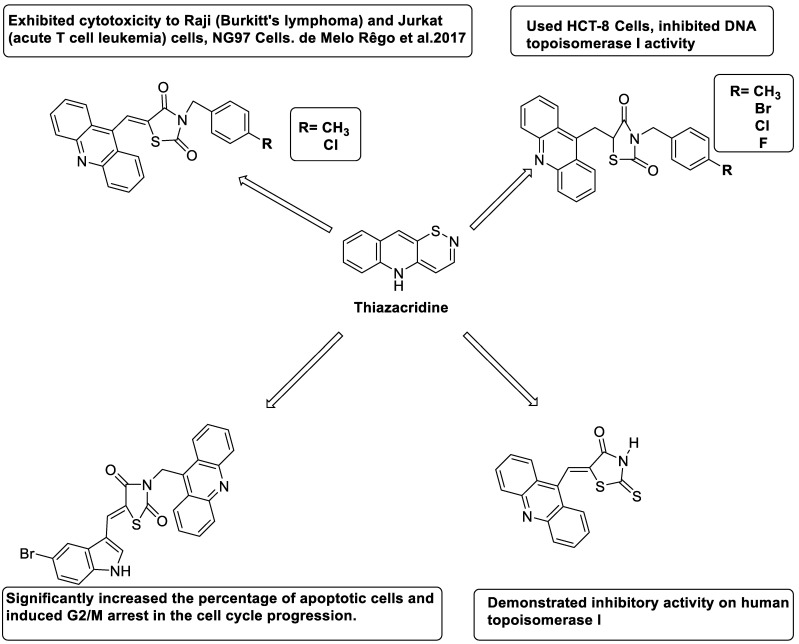
SAR of thiazacridine derivatives.

In several cancer cell lines, thiazacridine and imidazacridine derivatives have shown promise as tumour suppressors. That was the case in binding studies of 5-acridin-9-ylmethylidene-3-amino-2thioxo-thiazolidin-4-one, 5-acridin-9-ylmethylidene-2-thioxo-thiazolidin-4-one, 5-acridin9-ylmethylidene-2-thioxo-imidazolidin-4-one, and 3-acridin-9-ylmethylidene-2-thioxo-imid, except for 5-acridin-9-ylmethylidene-2-thioxo-1,3-thiazolidin-4-one, which showed inhibitory activity against human topoisomerase I. These findings shed light on the process by which imidazacridines and thiazacridines bind to DNA (Table 14 and Figure 12) [95].

### 2.15. Azacridine

**Figure 13:**
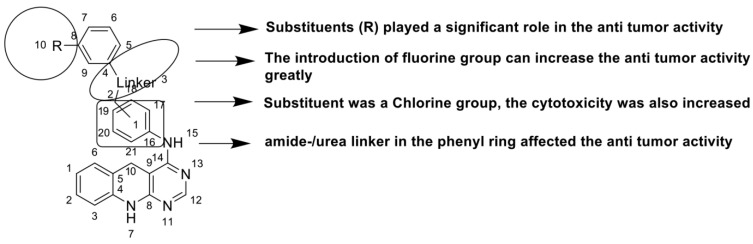
SAR of azaacridine derivatives as dual-target EGFR and Src kinase inhibitors.

A new category of azaacridine compounds, (Figure 13), which are effective EGFR and Src dual inhibitors, was designed and synthesised. According to the findings, the majority of the azaacridine compounds synthesised had good anti-proliferative activity against K562 and A549 cells. The representative 13b inhibited EGFR and Src activity, as well as tumour-cell invasion and apoptosis [96]. A variety of novel azaacridine analogues with a basic side chain were produced and their anti-proliferative efficacy was tested. The compounds had minimal biological activity against three cancer cell lines when compared with their acridine equivalents. This was thought to be due to the compounds’ hydrolytic instability in aqueous conditions (Table 15 and Table 16) [97].

## 3. Conclusions

Since the 1980s, several acridine derivatives have been synthesised and tested for anti-cancer efficacy. In recent years, the effective synthesis and anti-cancer efficacy of these nitrogen-containing heterocyclic moieties has been a point of interest. However, it has failed to treat cancer and hence falls short of our expectations and needs. In the coming years, a significant increase is feasible, but it will be contingent on the specialised design of compounds that target a single receptor, enzyme, or protein, with a focus on minimising side effects and toxicity. It is necessary to synthesise more of these heterocyclic compounds that target enzymes in order to modulate illness conditions. To summarise, the development of compounds containing an acridine nucleus is an attractive and promising area of medicinal chemistry, and pharmacophores containing this heterocyclic ring have the potential to contribute to the discovery of new biologically active drugs.

## Figures and Tables

**Table 1:** Acridine as an anti-tumour agent.

Compound	Structure	Biological Activity	Reference
Propyl-AcrDTU	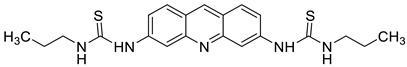	Leukaemia L1210 cells	[1]
Tetrandrine-based receptors	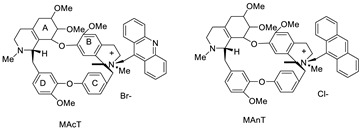	Anti-proliferative studies	[2]
9-(2′-hydroxyethylamino)-4-methyl-1-nitroacridine (C1748)	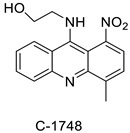	Phase II enzymes—UDP-glucuronosyltransferases (UGTs)	[3]
-	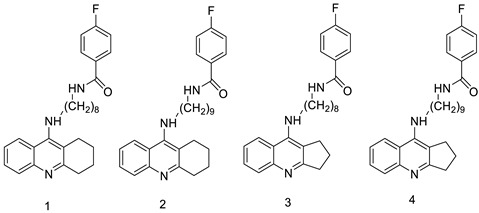	Human lung adenocarcinoma cells	[4]
Acridine chalcone 1C ((2 E)-3-(acridin-9-yl)-1-(2,6-dimethoxyphenyl)prop-2-en-1-one)	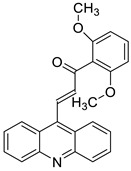	Human colorectal HCT116 cells	[5]
Novel spiro-acridine (E)-50-oxo-10-((3,4,5-trimethoxybenzylidene)amino)-10,50-dihydro-10H-spiro[acridine-9,20-pyrrole]40-carbonitrile (AMTAC-17)	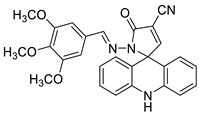	Anti-tumour activity of AMTAC-17	[6]
2-((6-Chloro-2-methoxy-acridin-9-yl)amino)-5,6,7,8-tetrahydro-4H-cyclohepta[b]-thiophene-3-carbonitrile (ACS03)	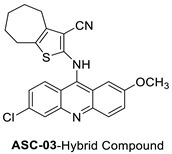	Anti-tumour activity	[7]
Dimethyl 2-[(acridin-9-yl) methylidene]-malonate (LPSF/IP-81)	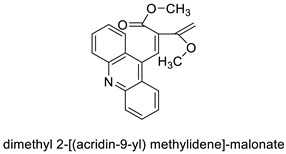	Invasive ductal carcinoma of human breast.	[8]
Bis (acridine-9-carboxylate)-nitro-europium (III) dihydrate	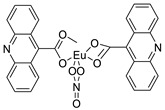	Anti-angiogenic and apoptotic activity against an animal model of carcinogenesis	[9]
9-amino-1-nitroacridine	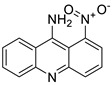	Pancreatic cancer	[10]
9-amino acridine derivatives	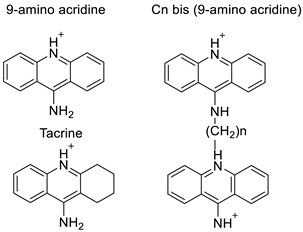	On alpha-adrenergic receptors	[11]
AT11-L0	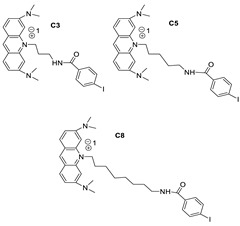	Anti-proliferative activity	[12]
Novel acridine-based N-acyl-homoserine lactone (AHL) analogs	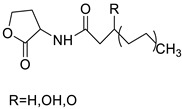	Human oral squamous carcinoma	[13]
New series of acridine hydroxamic acid derivatives	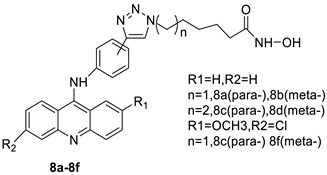	Topo II inhibition activity	[14]
9-chloro-2-(3-(dimethylamino) propyl) pyrrolo [2,3,4-kl] acridin-1(2H)-one (LS-1-10)	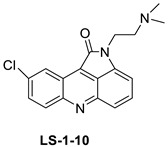	Colon cancer cells	[15]
Two iodinated acridine derivatives	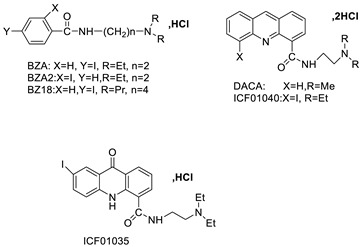	Targeted radionuclide therapy	[16]
[125I] ICF01035 and [125I] ICF01040	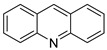	Melanoma-targeted in mice bearing B16F0	[17]
9-phenyl acridine (ACPH)	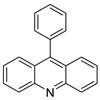	Anti-cancer agent	[18]
Tetra hydro acridine derivatives with iodobenzoic moiety	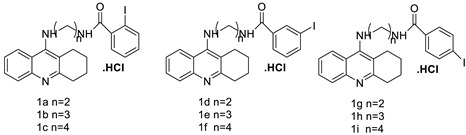	Human lung adenocarcinoma, human colorectal adenocarcinoma.	[19]
A series of 9-(2-(1-arylethylidene) hydrazinyl) acridine	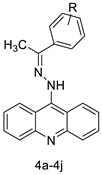	Anti-cancer activity	[20]
9-phenylacridine (ACPH)	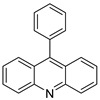	Antitumour activity	[21]
A class of acridine derivatives	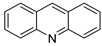	In vivo MM tumour growth	[22]
N0-(2-chloro-6-methoxy-acridin-9-yl)-2-cyano-3-(4-dimethylaminophenyl)-acrilohidrazida (ACS-AZ10). ACS-AZ10	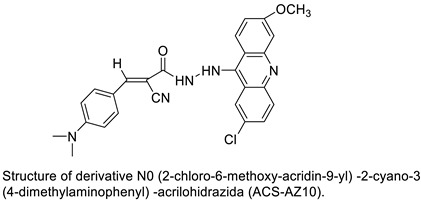	Anti-tumour activity	[23]
Acridine yellow G	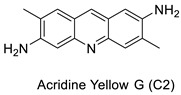	Inhibits both EGFR and PKCs	[24]
Unsymmetrical bisacridine derivatives (UAs)	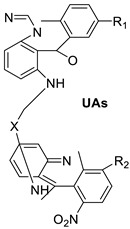	Cytotoxicity screening against several tumour cell lines	[25]
9-aryl-hexahydro-acridine-1,8-diones	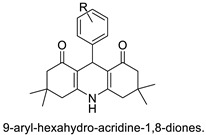	Anti-cancer activity against HepG2 and MCF-7 cell lines	[26]
Series of acridine-based catalytic inhibitors	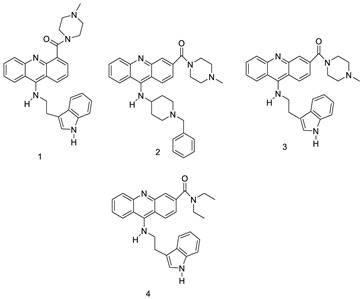	Pancreatic cancer and hTopoII catalytic inhibitors	[27]
Four hybrid acridine-HSP90	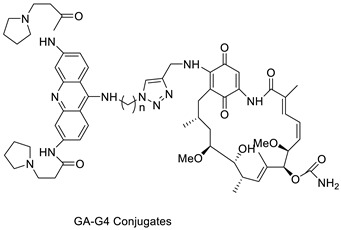	Telomerase inhibitor BRACO-19	[28]
Three new diphenyl substituted spiro triazolidine- and thiazolidinone-acridines	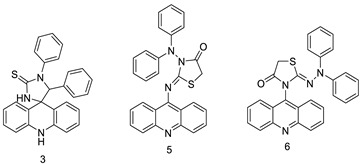	Interaction with calf thymus DNA	[29]
Amino acid appended acridines	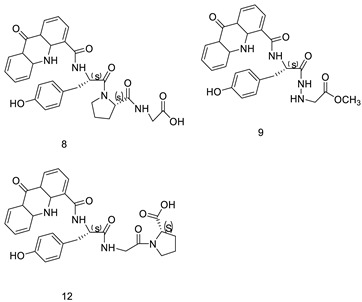	Anti-proliferative activity, arrested cells in G0/G1 phase of the cell cycle	[30]
A novel series of tri-substituted acridines	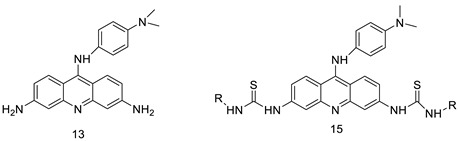	Mimicking the effects of BRACO19	[31]
DNA–polymer hybrids	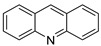	DNA–polymerisation	[32]
A novel DNA-cleaving agent CuGGHK-Acr	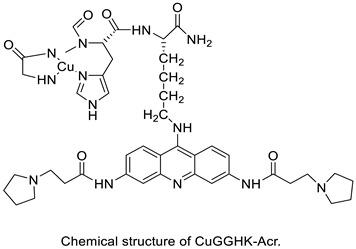	Targets G4 telomeric DNA	[33]
A series of 9-benzyl acridine derivatives	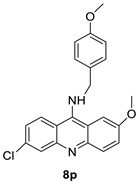	Anti-proliferative inhibitors	[34]

**Table 2:** 9-aminoacridine as an anti-tumour agent.

Compound	Structure	Biological Activity	Reference
Acridine-based catalytic inhibitors	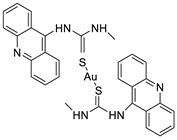	Anti-proliferative activity	[35]
CK0403	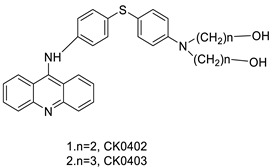	Treatment of breast cancer	[36]
9-amino acridines	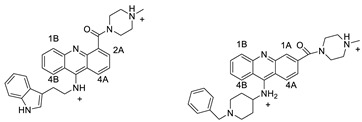	Anti-cancer activity	[37]
9-aminoacridine and artemisinin–acridine	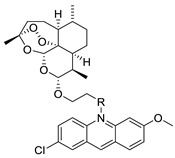	Anti-malarial activity, anti-cancer activity	[38]
9-aminoacridine (9AA)	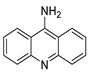	Anti-leukaemic cells	[39]
9-aminoacridine carboxamide	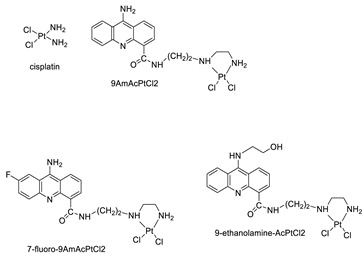	DNA-targeted intercalator	[40]
9-aminoacridine derivatives	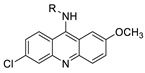	Anti-proliferative activity	[41]
Four acridine Pt complexes	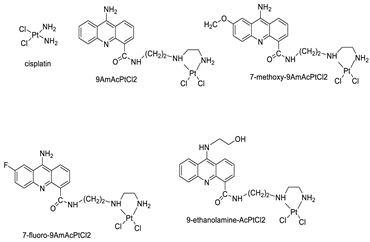	Detailed DNA sequence specificity	[42]
Small library of substituted 9-aminoacridine derivatives	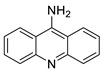	Ability to inhibit proliferation and induce cellular death in SCLC	[43]

**Table 3:** 9-anilinoacridine as an anti-tumour agent.

Compound	Structure	Biological Activity	Reference
2-methyl-9 substituted (AS 0–8) acridines	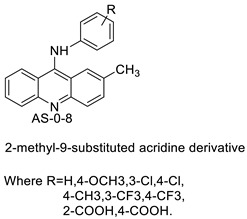	Lung carcinoma, breast cancer.	[44]
CK0403	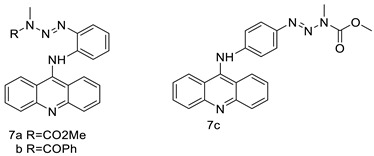	Anti-cancer activity	[45]
New series of 9-anilinoacridines containing phenyl-urea moieties	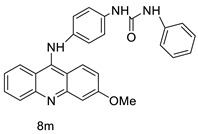	Novel dual Src and MEK inhibitors.	[46]
BO-1051	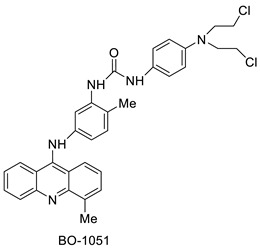	Against oral cancer	[47]
Novel isoxazole substituted 9 –anilinoacridines	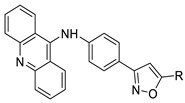	HER2 inhibitors.	[48]

**Table 4:** Acridine Thiourea gold as an anti-tumour agent.

Compound	Structure	Biological Activity	Reference
Two new 1-acridin-9-yl-3-methylthiourea	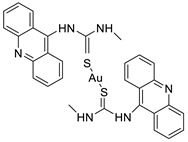	Human ovarian carcinoma cisplatin-sensitive A2780 cell line, breast cancer cell lines	[49]
Seven new cyclometalated Au (III) complexes	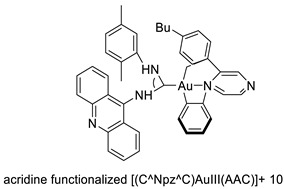	Cancer cells including leukaemia, lung and breast cancer cells	[50]

**Table 5:** Acridine-thiazolidinone as an anti-tumour agent.

Compound	Structure	Biological Activity	Reference
Three new acridine–thiazolidinone derivatives	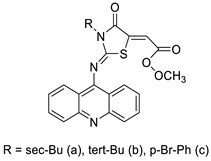	Leukemic cells, human epithelial ovarian cancer cell lines.	[51]
Series of novel hybrid 5-acridin-9-ylmethylene-3-benzyl-thiazolidine-2,4-diones	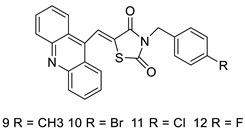	DNA interaction assays were performed	[52]

**Table 6:** Acridinone as an anti-tumour agent.

Compound	Structure	Biological Activity	Reference
Aseries of acridinones inspired by the structure of podophyllo toxin	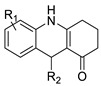	Triple-negative breast cancer cell line MDA-MB-231	[53]
A new series of 9(10H)-acridinone-1,2,3 triazole derivatives	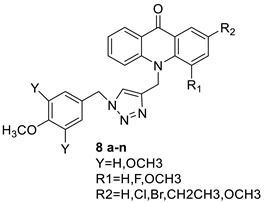	Against human breast cancer cell lines.	[54]
C-1305 C-1311	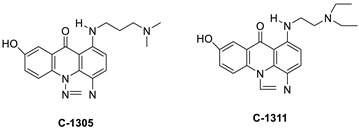	Anti-tumour agents	[55]

**Table 7:** Benzimidazole as an anti-tumour agent.

Compound	Structure	Biological Activity	Reference
New benzimidazole acridine derivative	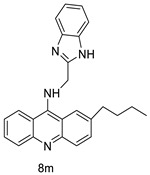	Human colon cancer cell lines	[56]
A series of 4-amidobenzimidazole acridines	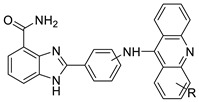	Inhibitory activities against Topo and PARP-1	[57]
Novel benzimidazole acridine derivatives	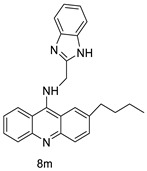	New DNA-targeted compounds	[58]

**Table 8:** Benzoacridine as an anti-tumour agent.

Compound	Structure	Biological Activity	Reference
Tubulin polymerisation inhibitors	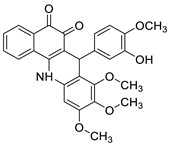	Eight cancer cell lines including MCF-7, A2780, HeLa, HepG2, DU145, A549, PC3, and LNCAP	[59]
New 7-substituted-5, 6-dihydrobenzo[c]acridine derivatives	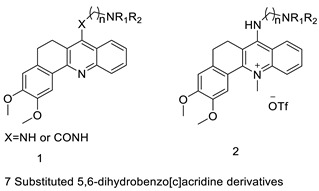	Induce apoptosis through activation of the caspase-3 cascade pathway	[60]
Phenanthrene fused-tetra hydrodibenzo-acridinones.	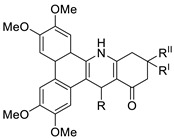	Cervical (HeLa), prostate (PC-3), fibrosarcoma (HT-1080), ovarian (SKOV-3) cancer cells	[61]
12-arylbenzoacridines	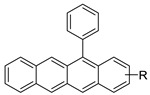	Selective estrogen-receptor modulators (SERMs).	[62]

**Table 9:** Imidazoacridinone as an anti-tumour agent.

Compound	Structure	Biological Activity	Reference
Imidazoacridinone C-1311	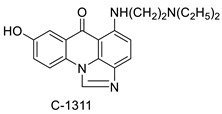	Anti-cancer activity	[63]
A novel photo activation-based pharmacological Trojan horse approach	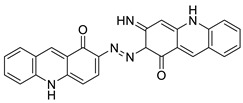	MDR tumour cells	[64]
C-1311	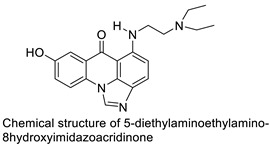	Anti-tumour agent C-1311in hepatoma cells	[65]
Crystal structures of NQO2 containing two of the imidazoacridin-6-ones	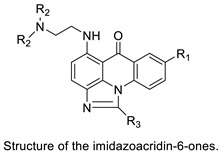	Inhibits the enzymatic function of NQO2 in cells.	[66]
Imidazoacridinone and triazoloacridinone	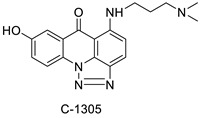	Liver cancer	[67]
C-1311	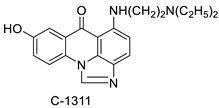	Human non-small-cell lung cancer (NSCLC) cell lines, A549 and H460. In A549 cells	[68]
C-1311 and C-1330	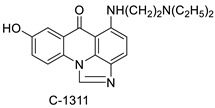	Human live microsomal enzymes	[69]
Imidazoacridinone C-1311	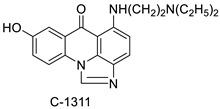	Against FLT3-ITD kinase	[70]

**Table 10:** Nitroacridine as an anti-tumour agent.

Compound	Structure	Biological Activity	Reference
C-1748, 9-(20-hydroxyethylamino)-4-methyl-1-nitroacridine	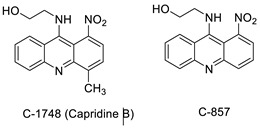	Anti-tumour activity	[71]
3-nitroacridine derivatives	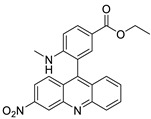	DNA-binding affinity and good inhibitory effect of tumour cell proliferation	[72]
New series of 1,3-dimethyl-6-nitroacridine derivatives	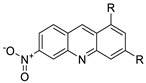	Anti-cancer activity against four cancer cells	[73]

**Table 11:** Oxazine as an anti-tumour agent.

Compound	Structure	Biological Activity	Reference
A series of 9-anilinoacridines substituted with oxazine derivatives	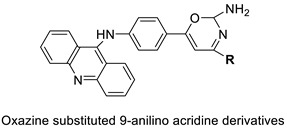	Antioxidant and anti-cancer activity	[74]
Novel oxazine substituted 9-anilinoacridines	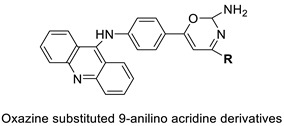	Topoisomerase II	[75]
A series of oxazine substituted 9-anilinoacridines	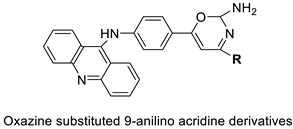	Anti-tumour activity on Dalton’s lymphoma ascites cells	[76]

**Table 12:** Platinum-acridine as an anti-tumour agent.

Compound	Structure	Biological Activity	Reference
Non-classical platinum-acridine hybrid agents	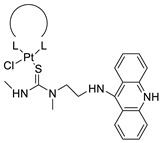	Distinct modules of DNA repair	[77]
A novel pharmacophore comprising a DNA-targeted platinum–acridine hybrid agent	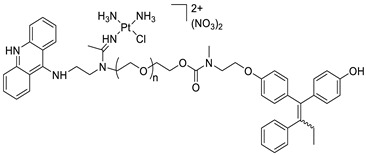	Breast cancer cell lines	[78]
P3A1	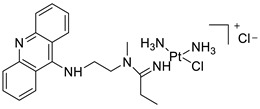	Cancer cells using MWCNTs as a drug carrier	[79]
[Pt(en)(L/L0)](NO3)2 [PtCl(en)(LH/L0H)](NO3)2	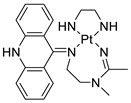	Inhibits H460 lung cancer cell proliferation	[80]
Platinum−acridine hybrid agents	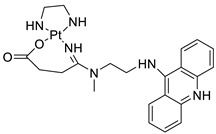	Anti-cancer activity	[81]
Platinum acridine hybrid agent [PtCl(en)(L)](NO3)2	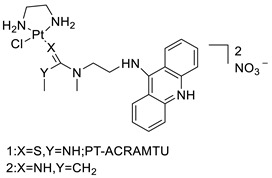	Delineate mechanistic differences between the platinum acridine hybrid agent	[82]
7-aminobenz[c] acridine	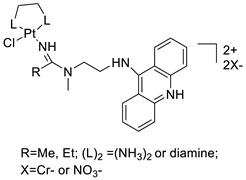	Tested in five NSCLC cell lines	[83]
Platinum–acridine agent	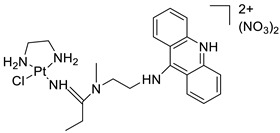	Structurally characterised	[84]
Platinum-acridine anti-cancer agent [PtCl(en)(LH)](NO3)2	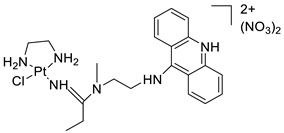	Chemo resistant non-small-cell lung cancer (NSCLC) cell lines	[85]

**Table 13:** Quinacrine as an anti-tumour agent.

Compound	Structure	Biological Activity	Reference
Quinacrine	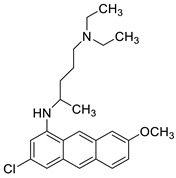	Human leukaemia K562cells	[86]
Quinacrine	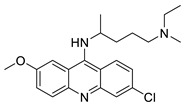	Human leukaemia U937 cells.	[87]
curcumin (Cur) and quinacrine (QC)	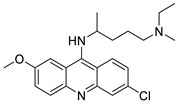	Induced breast epithelial transformed (MCF-10A-Tr) generated metastatic cells	[88]
Quinacrine	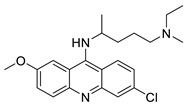	Inhibition of PARP-1PARylation	[89]
Quinacrine	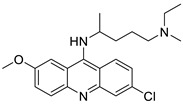	Cytotoxic effect of QC on DLBCL cells	[90]
Quinacrine	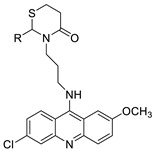	Against different cancer cell types	[91,92]

**Table 14:** Thiazacridine as an anti-tumour agent.

Compound	Structure	Biological Activity	Reference
Three new thiazacridine derivatives	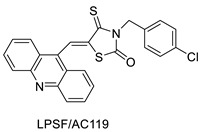	Induced neoplastic cell death primarily by apoptosis	[93]
An acridine and thiazolidine nucleus	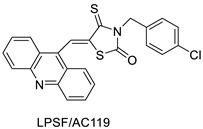	Tested in human colon carcinoma HCT-8 cells	[94]
Series of new thiazacridine agents	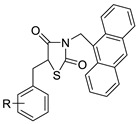	HepG2 cells	[95]
Thiazacridine and imidazacridine derivatives	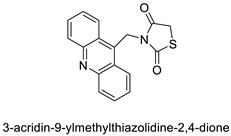	Tumours suppressors in some cancer cell lines	[96]

**Table 15:** Azaacridine as an anti-tumour agent.

Compound	Structure	Biological Activity	Reference
A new series of azaacridine derivatives	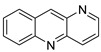	Potent EGFR and Src dual inhibitors	[97]
A series of new aza-acridine analogues	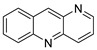	Anti-tumour agent	[97]

**Table 16:** Some of the most potent compounds and their IC_50_ (micromolar) values with cell lines.

Potent Compound	Cancer Cell Lines	Control	IC_50_ (µM)	Reference
Propyl-AcrDTU	HL-60	-	7.2–8	[1]
MAnT	HeLa cervical cancer cell line	Tetrandrine	2.74	[2]
C-1748	HT29 Cell	Sorafenib	39.7	[3]
Cmpd-**1**	A549 Cells	Etoposide	15	[4]
Cmpd-**2**	A549 Cells	“	10	
Cmpd-**3**	A549 Cells	“	15	
Cmpd-**4**	A549 Cells	“	10	
ACSO3	HCT-116 Cells	Doxorubicin	23.11 ± 1.03	[7]
	HeLa	“	˃50	
	MCF-7	“	˃50	
	K562	“	˃50	
	HL-60	“	˃50	
	HaCat	“	62.18 ± 1.15	
	PBMC	“	115.2 ± 5.82	
Cisplatin	MCF-7	-	12.7 ± 2.2	[9]
9-Acridine carboxylic acid	“	-	21.5 ± 3.1	
Eu(***III***)-complex	“	-	14.3 ± 2.6	
C-1748	MiaPaCa-2	-	0.015	[10]
	AsPC-1	-	0.075	
B1	SAS Cells	-	1.5	[13]
B2	“	-	11.7	
B3	“	-	5.3	
B4	“	-	18.0	
B5	“	-	9.4	
80c	U937 HCT-116	m-AMSA	0.90	[14]
LS-1-10	DLD1 Cells	Amsacrine	1.31	[15]
ACPH	A375	Camptothecin	20.74	[18]
1i	A549 HT-29	Etoposide	59.12–14.87 17.32–5.90	[19]
4b,4d,4e	HeLa, HepG2, HEK 239	Camptothecin	18.89–52.64 18.7–108.34 31.38–277.13	[20]
C2	malignant glioma and other human cancers.	erlotinib	0.75–5	[24]
C-1311	DU-145 HCT-116 MBA-MB-231	Gemcitabine Erlotinib	0.01–0.03 0.01–0.03 0.01–0.03	[25]
4b 4f 4g 4i 4j	HepG2 MCF-7	Doxorubicin	1.4 ± 0.11 4.7 ± 0.09 2.2 ± 0.09 5.3 ± 0.16 4.8 ± 0.12 4.4 ± 0.10 2.6 ± 0.11 5.9 ± 0.15 1.6 ± 0.14 5.0 ± 0.18	[26]
SR374 SR375 SR361 SR362	MCF7 A549 GIST48 WI38	-	1.6, 0.4, 2.9, <0.1 0.3, 0.1, 0.5, 0.1 1.5, 1.6, 2.8, 2.3 7.4, 4.5, 10.3, 0.7	[28]
Cmpd **5**	HL-60	Acridine	25.87 32.16	[29]
Cmpd **13** Cmpd **15** Cmpd **16** Cmpd **17** Cmpd **18**	HT-29	BRACO19	13.17–12.55 41.12–27.38 69.99–22.12 48.80–23.25 44.89–33.23	[31]
CuGGHK-Acr GGHK-Acr Ho-Acr	HuH-7 MCF-7 Caco2	-	9.8 ± 2.3 26.6 ± 2.2 16.7 ± 1.6	[33]
8p	A549 Cells	Doxorubicin Etoposide Amsacrine	0.61 ± 0.06	[34]
9AmAcPtCl2	HeLa Cells	Cisplatin	0.4	[40]
7r	HepG-2 MCF-7	Colchicin	4.2 23.8	[41]
7b	H1299 WM264 HCT116	Cisplatin	2.9 0.8 15.4	[45]
8m	K562 HepG-2	Imatinib	4.08 ± 0.14 9.41 ± 1.09	[46]
BO-1051	SAS Cells OECM1	-	2.39 1.97	[47]
Cmpd **2**	A2780 MDA-MB-231 SK-BR-3 MCF-7	CDDP AuC1PPh3	0.88 ± 0.20 2.75 ± 0.40 3.62 ± 0.14 5.32 ± 0.65	[50]
Complex **11**	A549 MCF-7 HL60	Cisplatin	7.6 ± 0.6 1.5 ± 0.1 1.1 ± 0.1	[50]
2C	HL-60 L1210 A2780	Cisplatin	1.3 ± 0.2 3.1 ± 0.4 7.7 ± 0.5	[51]
Cmpd-**9**	PC-3 COLO-205	Amsacrine	5.5–9.5 8.6–42.3	[52]
Cmpd-**6** Cmpd-**7** Cmpd-**9** Cmpd-**10**	MDA-MB-231 DU-145	Colchicine	3 ± 1, 3.2 ± 0.7 0.190 ± 0.007 1.1 ± 0.2 1.0 ± 0.3 0.11 ± 0.02 0.12 ± 0.02	[53]
8c	MCF-7 T-47D MDA-MB-231	etoposide	11 ± 4.8 14.5 ± 5.2 16.6 ± 5.9	[54]
C-1305 C-1311	KB-3	-	0.263 ± 0.016 0.106 ± 0.014	[55]
8m	SW480 HCT116	-	6.77 ± 0.19 3.33 ± 0.02	[56]
11L	PARP-1 MCF-7	Olaparib m-AMSA	0.45 ± 0.03 2.14 ± 0.92	[57]
8I	K562 HepG-2	Colchicine Imatinib	2.68 8.11	[58]
4g	HUVEC LNCAP	Β-Lapachone	49.19 ± 2.3 18.54 ± 2.11	[59]
2b	HeLa K562 A549	-	3.2 9.2 5.7	[60]
8m	SKOV-3	Cisplatin	0.24 ± 0.05	[61]
Cmpd-**1**	MCF-7	Tamoxifen citrate	0.951	[62]
6a1	HCT116	NQO2	14 ± 4	[66]
C-1311	MV4-11 MOLM13	-	0.03 ± 0.01 0.04 ± 0.02	[70]
Cmpd-**2**	MCF-7 MDA-MB-231 SGC7901 MGC803	-	6.79 ± 2.01 6.46 ± 2.19 17.25 ± 3.84 10.94 ± 2.26	[72]
Cmpd-**1** Cmpd-**6**	MCF-7 MDA-MB-231 SGC7901 MGC803	-	8.83 ± 0.98 10.02 ± 0.78 41.47 ± 3.24 23.96 ± 1.54	[73]
5a	DLA Cells	ascorbic acid	20.03 ± 0.2583	[74]
8	MCF-7 MDA-MB-231	Tamoxifen	1.6 ± 0.4 13.2 ± 0.1	[78]
Cmpd-**3**	NCI-H460 OVCAR-3 PANC-1	Cisplatin	0.008 ± 0.002 1.1 ± 0.1 0.09 ± 0.01	[80]
3a 3b	H460	-	12 ± 2 2.8 ± 0.3	[81]
P1-A1 P1-B1 P1-B2	NCI-H460	-	0.0052 ± 0.0001 0.24 ± 0.01 2.4 ± 0.5	[83]
Cmpd-**1**	NCI-H460 NCI-H522	Cisplatin	8 ± 2 18 ± 2	[85]
QC	OCI-Ly01 SU-DHL-8	Quinacrine	1.8 2	[90]
Cmpd-**25**	MDA-MB-468 MDA-MB-231 MCF-7 184B5	Cisplatin Quinacrine	1.73 ± 0.80 2.80 ± 1.30 0.69 ± 0.41 4.96 ± 0.24	[91]
Cmpd-**11**	MDA-MB-468 MDA-MB-231 MCF-7 184B5	Cisplatin Quinacrine	2.40 ± 1.01 1.92 ± 0.20 1.24 ± 0.51 16.16 ± 0.81	[92]
LPSF/AC-119	Raji Jurkat	Amsacrine	0.6 1.53	[93]
AC-4 AC-7 AC-10 AC-23	HCT-8	Amsacrine	3.1 5.3 3.6 2.3	[94]
7a 7b	HepG2	Amsacrine	68.03 48.63	[95]
13b	K562 A549	Imatinib	0.22 ± 0.03 0.253 ± 0.16	[97]

## Data Availability

The data presented in this study are available on request from the corresponding author.
